# Occlusion-Aware Trajectory Discontinuity Correction for Roadside LiDAR Using Time–Space Analysis

**DOI:** 10.3390/s26123755

**Published:** 2026-06-12

**Authors:** Mingshu Dong, Hao Xu, Muchen Tian, Fei Guan, Ziru Wang, Renjuan Sun, Yanhua Guan

**Affiliations:** 1 Department of Civil and Environmental Engineering, University of Nevada, Reno, Reno, NV 89557, USA; mdong@unr.edu (M.D.); muchent@unr.edu (M.T.); fguan@unr.edu (F.G.); ziruw@unr.edu (Z.W.); 2 School of Qilu Transportation, Shandong University, Jinan 250002, China; sunrenjuan@sdu.edu.cn (R.S.); guanyanhua@sdu.edu.cn (Y.G.)

**Keywords:** roadside LiDAR, vehicle trajectory, occlusion, trajectory discontinuity, time–space analysis, traffic monitoring, post-processing

## Abstract

Recent advances in roadside sensing technologies, including camera-based systems, radar, and LiDAR, have enabled high-resolution sampling of vehicle trajectories, overcoming the temporal and spatial limitations of traditional data collection methods. Among these, LiDAR sensing has been widely adopted for traffic monitoring and surrogate safety analysis due to its high spatial accuracy and temporal resolution. However, sensor noise and occlusion in roadside LiDAR frequently introduce tracking point offsets and trajectory discontinuities, reducing the reliability of vehicle counts, traffic state estimation, and conflict analysis. To address these challenges, this study proposes a post-processing method based on time–space analysis to detect and correct occlusion-induced trajectory discontinuities. By exploiting the inherent spatiotemporal consistency of vehicle movements, the proposed approach identifies fragmented trajectories, reconstructs continuous vehicle paths, and recovers realistic traffic patterns. Validated on real-world LiDAR data collected at an urban intersection in Reno, Nevada, across four 30 min traffic periods covering AM and PM peak conditions on weekdays and weekends, the proposed method achieves an average precision of 0.989 and an average F1-score of 0.948, outperforming IMM, GNN-RM, and HMM + Viterbi benchmark methods. Count accuracy improved from 85.5% to 97.4% across all evaluated periods, confirming the method’s effectiveness under occlusion conditions.

## 1. Introduction

Traffic systems are inherently complex and dynamic, involving interactions among vehicles, infrastructure, and human behavior. Understanding traffic movements and interactions at a microscopic level is essential for applications such as safety analysis, infrastructure design, and intelligent transportation systems. In recent years, vehicle trajectory data, which describe the continuous movement of individual vehicles in space and time, have emerged as a critical data source for such analyses. Compared with traditional traffic data (e.g., loop detector counts or speeds), trajectory data provide richer spatiotemporal information, enabling detailed investigation of vehicle interactions, queue formation, and traffic dynamics [1,2,3].

Trajectory data can generally be obtained from probe-based methods or roadside sensing systems. Probe-based data, such as those collected from GPS-enabled vehicles or mobile devices, provide wide spatial coverage but are often limited by low penetration rates and positioning inaccuracies. In contrast, roadside sensing technologies enable direct observation of traffic participants within a localized detection area, offering high resolution and high penetration rate trajectory data that are particularly suitable for microscopic traffic analysis [4].

Among roadside sensing technologies, cameras, radar, and LiDAR each offer distinct advantages and limitations for trajectory data collection. Camera-based systems provide rich semantic information and can detect and classify multiple types of road users; however, their performance is sensitive to lighting and weather conditions, and geometric calibration is required to convert image coordinates into real-world spatial positions. Radar sensors are robust under adverse weather conditions and can directly measure vehicle speeds via the Doppler effect; however, they are limited by relatively low spatial resolution and a reduced ability to capture detailed object geometries. LiDAR sensors offer high spatial accuracy, fine-grained temporal resolution [3], and a wide field of view, enabling detailed geometric characterization of traffic participants and high-quality trajectory data collection [5]. In this study, LiDAR sensing is adopted for trajectory data collection, as its spatial and temporal resolution better satisfies the requirements of microscopic traffic monitoring tasks.

Despite these advantages, generating accurate and reliable trajectory data from roadside LiDAR remains challenging. In real-world environments, occlusion, sensing noise, and environmental disturbances frequently degrade the quality of point cloud data. Among these factors, occlusion is particularly critical, as vehicles may be temporarily blocked by surrounding objects, leading to missing observations or fragmented trajectory segments. Such issues can result in trajectory discontinuities, tracking point offsets, and misinterpretation of vehicle movements. Consequently, such errors may propagate into downstream applications, causing biased traffic movement analysis, inaccurate vehicle counts, and unreliable safety assessments [6].

This study proposes an occlusion-aware trajectory discontinuity correction framework based on time–space analysis, addressing a critical yet underexplored challenge in roadside LiDAR-based traffic monitoring. The key innovation lies in a unified spatiotemporal approach that combines occlusion-aware endpoint prediction with a spatiotemporal consistency-based matching and stitching strategy to reconstruct fragmented vehicle trajectories. Unlike existing methods that rely on motion model assumptions or require large training datasets, the proposed framework operates directly on trajectory-level spatiotemporal representations without requiring additional sensor input or model training. Validated on real-world LiDAR data at an urban intersection in Reno, Nevada, across four 30 min peak-period datasets covering AM and PM conditions on weekdays and weekends, the method achieves an average precision of 0.989 and an average F1-score of 0.948, outperforming IMM, GNN-RM, and HMM + Viterbi benchmark methods.

The remainder of this paper is organized as follows. Section 2 reviews existing studies on trajectory enhancement and reconstruction. Section 3 presents the proposed methodology. Section 4 describes the experimental setup and reports the validation results. Section 5 concludes the study and discusses future research directions.

## 2. Literature Review

Trajectory enhancement and reconstruction methods have been extensively studied to address data incompleteness and improve trajectory data quality. This section reviews three relevant bodies of work: (1) motion-model-based and constraint-based reconstruction, (2) tracking and data association-based methods, whose limitations under occlusion motivate the present study, and (3) spatiotemporal analysis-based approaches.

### 2.1. Motion-Model-Based and Constraint-Based Methods

A substantial body of research has focused incomplete trajectories by leveraging vehicle motion models or trajectory-level physical constraints. These methods primarily rely on physical or behavioral assumptions to infer missing trajectory segments, rather than directly linking observations.

For example, Luo et al. [7] proposed a trajectory repair framework for roadside LiDAR data under full occlusion and limited observations, where candidate trajectory segments are reconnected based on vehicle interaction constraints, such as spacing and motion consistency, while Kalman filtering is incorporated for state estimation, including tracking position, velocity, and acceleration, and trajectory smoothing. Similarly, earlier studies by Herrera et al. [8] and Hunter et al. [9] explored trajectory inference from sparse probe data using statistical and probabilistic models, providing a foundation for model-driven reconstruction approaches.

Beyond Kalman-based smoothing, car-following models and optimization-based methods also fall within this category, as both rely on physical or behavioral constraints to infer missing states. Car-following models estimate unobserved vehicle states based on leader–follower interactions, assuming consistent driving behavior. Optimization-based approaches similarly enforce physical consistency: Wang et al. [10] formulated trajectory reconstruction as a minimum-cost flow problem, where candidate trajectory segments are connected by minimizing a global cost function under feasibility constraints.

Macroscopic traffic theory has further been incorporated into trajectory reconstruction. Chen et al. [11] utilized shockwave theory to reconstruct vehicle trajectories by identifying traffic wave propagation patterns in the time–space domain. Similarly, Zhang et al. [12] proposed a time-varying shockwave speed model to better capture dynamic traffic conditions.

Although these methods provide strong physical interpretability, they rely heavily on assumptions regarding vehicle movement consistency or driver behavior. Their performance may degrade in heterogeneous traffic environments, near intersections with complex maneuvers, or under noisy sensing conditions where such assumptions are difficult to maintain.

### 2.2. Tracking and Data Association-Based Methods

In contrast to model-based approaches, a separate line of research addresses trajectory continuity from a tracking perspective, aiming to prevent fragmentation rather than correct it after the fact. Since trajectory enhancement is typically a post-processing step, the quality of upstream tracking directly determines the degree of fragmentation that downstream enhancement methods must address. This section therefore reviews tracking and data association methods, highlighting their limitations under occlusion as motivation for the post-processing approach proposed in this study. Classical tracking frameworks include Global Nearest Neighbor (GNN), Multiple Hypothesis Tracking (MHT), and Joint Probabilistic Data Association (JPDA), often combined with Kalman filtering for state estimation.

Recent studies have further improved tracking performance. Lin et al. [13] applied the Hungarian algorithm to match detected vehicles based on spatial proximity and motion similarity, improving short-term tracking performance. Deng et al. [14] leveraged multi-sensor roadside observations to enhance trajectory reconstruction through improved data association and sensor fusion.

Deep learning-based tracking methods have also been explored, where object detection and tracking are jointly optimized using neural networks (e.g., PointNet-based and multi-view detection frameworks). These approaches improve robustness to noise and detection errors, particularly in complex traffic environments.

However, tracking-based methods primarily rely on short-term temporal consistency and frame-to-frame associations. When occlusion occurs or observations are missing for extended durations, these methods are prone to identity switching, trajectory fragmentation, and tracking loss. Consequently, their ability to recover long-duration trajectory discontinuities remains limited.

### 2.3. Spatiotemporal Analysis-Based Methods

To overcome the limitations of constrained methods, recent research has explored trajectory reconstruction from a spatiotemporal perspective. In time–space representations, vehicle trajectories form structured patterns that reflect underlying traffic dynamics, enabling more global reasoning for trajectory reconstruction.

Probabilistic approaches have also been developed. Mei et al. [15] introduced a Bayesian framework to estimate traffic states using probe vehicle trajectories in the time–space domain. Seo et al. [16] provided a comprehensive review of traffic state estimation techniques, emphasizing the role of spatiotemporal modeling.

More recent studies have focused on LiDAR-based trajectory reconstruction. Chang et al. [17] proposed a spatiotemporal stacking method that improves tracking robustness by leveraging temporal consistency across multiple frames. Gao et al. [18] enhanced trajectory extraction accuracy through improved clustering and denoising techniques, thereby indirectly supporting trajectory continuity.

Despite these advances, existing spatiotemporal approaches still face challenges under severe occlusion and highly fragmented trajectories. Many methods rely on global optimization or statistical inference, which may inadequately capture local physical constraints or demand extensive parameter calibration.

## 3. Proposed Methodology

### 3.1. Time–Space Diagram Characteristics

In the time–space diagram, the horizontal axis represents the frame index (or elapsed time), and the vertical axis represents the longitudinal position of a vehicle within the detection zone. Each polyline corresponds to a single detected trajectory segment, while individual markers denote the point-level LiDAR observations from which the segment is derived. Under nominal conditions, a vehicle traversing the detection zone is expected to produce a temporally ordered and spatially continuous curve exhibiting a physically plausible slope. Deviations from this expected pattern, including abrupt truncations, local geometric deformations, or identity inconsistencies, are indicative of errors in the upstream tracking output. Four representative trajectory anomaly types are identified in this study: full occlusion, partial occlusion, trajectory distortion and ID switching.

**Figure 1:**
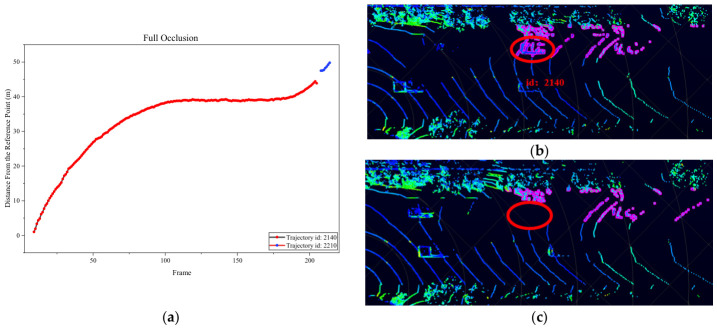
Full occlusion: (**a**) time–space diagram showing trajectory split; (**b**) point cloud prior to full occlusion (Frame 2108); (**c**) point cloud during full occlusion (Frame 2113). In the point cloud snapshots, purple points denote LiDAR returns within the defined detection zone.

For full occlusion and partial occlusion, described in Section 3.1.1 and Section 3.1.2, three subfigures are provided: Figure 1a presents the corresponding time–space diagram, and Figure 1b,c provide LiDAR point cloud snapshots of representative frames illustrating the anomaly, with the spatial extent of the detection zone highlighted in purple. For trajectory distortion and ID switching, described in Section 3.1.3, only the time–space diagrams are provided, as these anomaly types do not directly affect vehicle counts, and their point cloud illustrations are not essential to the primary contribution of the paper.

#### 3.1.1. Full Occlusion

Full occlusion arises when a target vehicle is completely obscured by a proximate object, typically a large vehicle such as a truck, for a sufficiently prolonged duration. As illustrated in Figure 1a, this phenomenon manifests in the time–space diagram as a discrete discontinuity in which a single vehicle trajectory is partitioned into two temporally separated segments. When the duration of missing observations exceeds the predefined temporal threshold, the tracking algorithm terminates the current trajectory; upon re-emergence of the vehicle, a new trajectory identifier is assigned, producing trajectory fragmentation and inflated vehicle counts.

#### 3.1.2. Partial Occlusion

**Figure 2:**
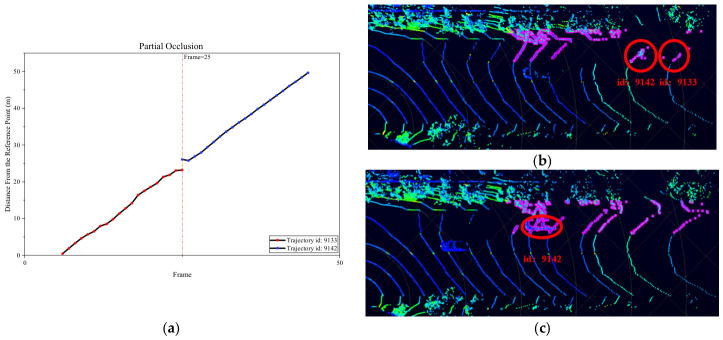
Partial occlusion: (**a**) time–space diagram showing trajectory duplication; (**b**) point cloud prior to partial occlusion (Frame 15,397); (**c**) point cloud during partial occlusion (Frame 15,411).

Partial occlusion occurs when only a portion of the target vehicle is obscured, causing the visible sections to generate two spatially distinct point cloud clusters corresponding to a single physical vehicle. The corresponding time–space diagram and point cloud snapshots are shown in Figure 2. In the time–space diagram, this condition is reflected as two parallel or temporally overlapping trajectory segments assigned distinct identifiers by the clustering and tracking algorithm. In contrast to full occlusion, which produces temporally disjoint segments, partial occlusion results in trajectory duplication with concurrent temporal overlap, which motivates the introduction of the overlap-based screening constraint presented in Section 3.5.3.

#### 3.1.3. Trajectory Distortion and ID Switching

**Figure 3:**
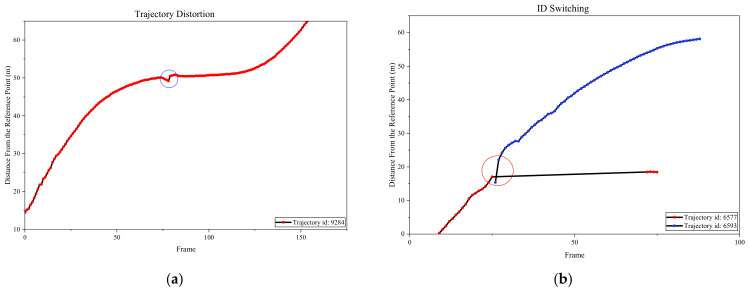
Time–space diagrams of trajectory anomaly types: (**a**) trajectory distortion showing oscillatory pattern; (**b**) ID switching showing identity transition.

Figure 3 illustrates representative instances of trajectory distortion and ID switching in the time–space diagram. Trajectory distortion denotes short-term geometric irregularities in the time–space representation attributable to transient instabilities in the LiDAR point cloud. When the forward portion of a vehicle becomes occluded, the computed centroid migrates toward the visible rear section; conversely, when the rear portion is occluded, the centroid shifts forward. ID switching occurs when the tracking algorithm fails to maintain a consistent object identity due to the close spatial proximity of two or more vehicles. Upon subsequent vehicle separation, the algorithm may assign a new identifier to the original vehicle instead of restoring the prior identity.

Considered collectively, full occlusion and partial occlusion are the primary sources of trajectory fragmentation and duplication, directly contributing to vehicle count errors. Trajectory distortion and ID switching, by contrast, predominantly affect the geometric integrity and identity continuity of trajectories without directly altering vehicle counts. These representative anomaly patterns demonstrate the diagnostic utility of time–space diagrams and motivate the discontinuity identification and reconstruction framework developed in the subsequent sections.

### 3.2. Overall Framework

**Figure 4:**
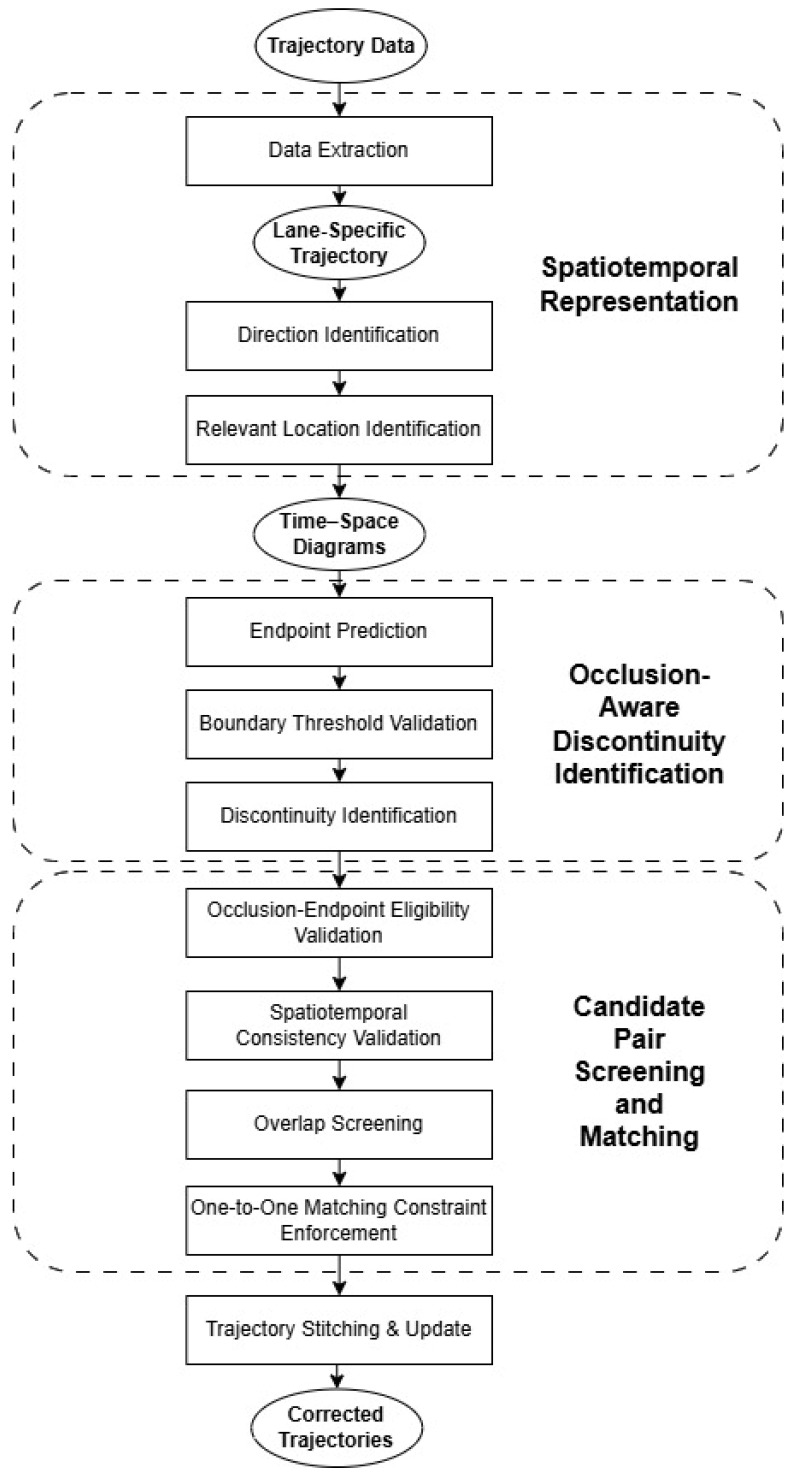
Framework of the proposed time–space analysis-based trajectory discontinuity correction method.

Figure 4 illustrates the framework of the proposed methodology. The process includes three main stages: spatiotemporal representation, occlusion-aware discontinuity identification, and candidate pair screening and matching. First, raw trajectory data are transformed into lane-specific trajectories and represented in time–space diagrams. Second, potential trajectory discontinuities caused by occlusion are identified through endpoint prediction and boundary threshold validation. Third, candidate trajectory segments are screened through occlusion eligibility validation, spatiotemporal consistency validation, overlap screening, and one-to-one matching enforcement. Finally, the matched trajectory segments are stitched together to generate corrected vehicle trajectories.

### 3.3. Spatiotemporal Representation

**Figure 5:**
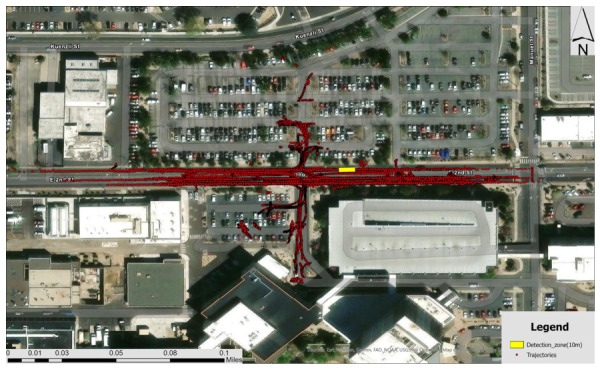
Vehicle trajectories and 10 m-long lane detection zone used for occlusion analysis.

Transforming raw LiDAR-derived trajectories into a unified spatiotemporal representation is a prerequisite for the systematic detection of trajectory discontinuities, as this representation makes explicit the geometric and temporal structure of vehicle movements that would otherwise remain obscured in raw point cloud data. Figure 5 illustrates the vehicle trajectories and 10 m-long lane detection zone employed in this study.

#### 3.3.1. Trajectory Preprocessing

The raw trajectory dataset is formally defined as:

(1) $$T=\left\{\tau_{i}|\tau_{i}={\left\{\left(t_{k},x_{k},y_{k}\right)\right\}}_{k=1}^{n_{i}}\right\}$$

where T denotes the complete set of trajectories; $\tau_{i}$ represents the *i*th trajectory; $n_{i}$ is the total number of observation points within trajectory $\tau_{i}$; $t_{k}$ is the timestamp associated with the *k*th point; and $x_{k}$, $y_{k}$ are the corresponding spatial coordinates. A semantic filtering operation is subsequently applied:

(2) $$T^{veh}=\left\{\tau_{i}\in T|\mathrm{class}\left(\tau_{i}\right)=\mathrm{vehicle}\right\}$$

where $T^{veh}$ denotes the filtered trajectory set restricted to vehicular road users, and class ($\tau_{i}$) specifies the object category assigned to trajectory $\tau_{i}$.

#### 3.3.2. Detection Zone Construction

Each detection zone is formally defined as:

(3) $$Z_{j}=\left\{\left(x,y\right)|\left(x,y\right)=R\left(\theta \right)\cdot \left(u,v\right)+c_{j},\;u\in \left[0,L_{j}\right],\;v\in \left[-\frac{W}{2},\frac{W}{2}\right]\right\}$$

where $Z_{j}$ denotes the *j*th detection zone; $c_{j}$ specifies the zone center point; u and v are local coordinates expressed in the zone reference frame; $L_{j}$ is the zone length; W is the zone width; θ is the rotation angle relative to the global coordinate system; and $R\left(\theta \right)$ is the corresponding 2D rotation matrix.

#### 3.3.3. Relevant Location Identification

The set of trajectory observations falling within a given detection zone is defined as:

(4) $$P_{ij}=\left\{p\in \tau_{i}|p\in Z_{j}\right\}$$

where $P_{ij}$ denotes the subset of points from trajectory $\tau_{i}$ located within zone $Z_{j}$, and p represents an individual trajectory observation. Consecutive observation points are connected to form trajectory segments:

(5) $$l_{k}=\|p_{k+1}-p_{k}\|$$

where $l_{k}$ denotes the Euclidean length of the *k*th segment and $\left\|p_{k+1}-p_{k}\right\|$ is the Euclidean distance operator. The cumulative in-zone travel distance is subsequently computed as:

(6) $$L_{ij}=\underset{k}{\sum }l_{k}\cdot 1\left(s_{k}\cap z_{j}\neq \emptyset \right)$$

where $L_{ij}$ is the total in-zone travel distance for trajectory $\tau_{i}$ within zone $Z_{j}$; $s_{k}$ denotes the *k*th segment; and 1(·) is the indicator function. To construct a consistent one-dimensional time–space representation, spatial coordinates are projected onto an axis aligned with the dominant direction of vehicle movement, with the reference boundary determined as:

(7) $$B=\arg\underset{B\in \left\{B_{1},\;B_{2}\right\}}{\max}\underset{\tau_{i}}{\sum }1\left(\frac{d_{B}\left(t_{end}\right)-d_{B}\left(t_{start}\right)}{t_{end}-t_{start}}>0\right)$$

where $B_{1}$ and $B_{2}$ denote the two spatial boundaries of the detection zone; $t_{start}$ and $t_{end}$ are the entry and exit timestamps of trajectory $\tau_{i}$; and 1(·) serves as an indicator of trajectory exhibiting a predominantly increasing distance trend. This automated direction identification procedure eliminates the need for manual specification of lane travel direction, providing a consistent spatial reference for subsequent discontinuity detection and trajectory reconstruction.

### 3.4. Occlusion-Aware Discontinuity Identification

This section presents the occlusion-aware discontinuity identification framework, comprising three sequential components: endpoint prediction, boundary threshold validation, and discontinuity classification.

#### 3.4.1. Endpoint Prediction

For each trajectory segment $\tau_{i}$, short-horizon motion prediction is performed at both the initial and terminal endpoints to estimate the probable continuation of the trajectory beyond the observed data. The trajectory segment is represented in the time–space domain as:

(8) $$\tau_{i}={\left\{\left(t_{k},\;s_{k}\right)\right\}}_{k=1}^{n_{i}}$$

where $t_{k}$ denotes the observation timestamp and $s_{k}$ denotes the corresponding spatial coordinate. The instantaneous velocity at each observation point is estimated as

(9) $$v_{k}=\frac{s_{k+1}-s_{k}}{t_{k+1}-t_{k}}$$

using a fixed sliding window of k+1 consecutive points. The predicted endpoint is defined according to the following piecewise formulation:

(10) $${\hat{S}}_{i}^{end}=\left\{\begin{array}{cc} s_{i}^{end}+\bar{v}\Delta t & ,if\;\forall k,\;v_{k}>0\\ \left[S_{min}+\Delta t,S_{min}+{\bar{v}}_{i}\Delta t\right] & ,otherwise \end{array}\right.$$

where ${\hat{s}}_{i}^{end}$ denotes the predicted endpoint position; ${\bar{v}}_{i}=\frac{1}{K}\sum v_{k}$ represents the mean velocity computed over the prediction window; Δt is the prediction horizon; and $S_{min}$, $S_{max}$ denote the minimum and maximum spatial values observed within the window. When all velocity estimates within the window are strictly positive, a deterministic point prediction is applied under the assumption of stable forward motion. Otherwise, an interval-based prediction is employed to accommodate local velocity fluctuations attributable to sensor noise or partial occlusion.

#### 3.4.2. Boundary Threshold Validation

To distinguish between occlusion-induced trajectory discontinuities and natural trajectory terminations arising from vehicles entering or exiting the detection zone, spatial threshold constraints are imposed on the effective spatial domain:

(11) $$s\in \left[\delta ,\;L-\delta \right]$$

where L denotes the length of the detection zone and δ is a prescribed boundary tolerance parameter. Trajectory endpoints whose predicted continuations fall outside this interior region are classified as natural terminations and excluded from subsequent discontinuity identification; only those endpoints with predicted continuations residing within the interior domain are retained as candidate occlusion-induced terminations.

#### 3.4.3. Discontinuity Identification

Based on the validated endpoint predictions, trajectory discontinuities are formally identified according to the criterion

(12) $$\tau_{i}\in D\;\Leftrightarrow \;\left(s_{i}^{end}<L-\delta \right)\vee \left(s_{i}^{start}>\delta \right)$$

where D denotes the set of trajectory segments classified as discontinuous. Within the time–space representation, trajectories exhibiting normal traversal behavior are expected to progress continuously across the detection zone. Occlusion-induced fragmentation, by contrast, produces truncated segments whose predicted continuations remain confined to the zone interior, providing a geometrically direct and physically interpretable criterion for discontinuity identification.

### 3.5. Candidate Pair Screening and Matching

The trajectory matching process is formulated as a constrained decision procedure in which a candidate segment pair is accepted for stitching only when a prescribed set of spatiotemporal and logical conditions is simultaneously satisfied. The framework comprises four sequential validation components.

#### 3.5.1. Occlusion-Endpoint Eligibility Validation

A candidate segment pair ($\tau_{i}$, $\tau_{j}$) is eligible for matching only if both the terminal endpoint of $\tau_{i}$ and the initial endpoint of $\tau_{j}$ satisfy the occlusion conditions established in Section 3.4:

(13) $$O_{i}^{trail}=1\;\wedge \;O_{j}^{head}=1$$

where $O_{i}^{trail\;}$ and $O_{j}^{head}$ denote the binary occlusion indicators for the tail and head endpoints of the respective segments. This constraint ensures that only trajectory segments showing evidence of occlusion-induced termination are considered for matching, thereby excluding spurious candidates.

#### 3.5.2. Spatiotemporal Consistency Validation

Spatiotemporal consistency between a candidate pair is assessed by jointly evaluating the temporal gap and spatial continuity between the two segments. The temporal separation is defined as:

(14) $$T_{min}\leq t_{j}^{start}-t_{i}^{end}\leq T_{max}$$

The spatial feasibility condition for point-based endpoint predictions is expressed as:

(15) $$S_{min}\leq {\hat{S}}_{i}^{start}-\;{\hat{S}}_{i}^{end}\leq S_{max}$$

For interval-based endpoint predictions, the spatial condition takes the form:

(16) $${\hat{S}}_{i}^{end}\in \left[a_{1},a_{2}\right],{\hat{S}}_{i}^{start}\in \left[b_{1},b_{2}\right],\;\;\exists \;S_{i}\in \left[a_{1},a_{2}\right],\;S_{j}\in \left[b_{1},b_{2}\right]:S_{j}-S_{i}\in \left[S_{min},S_{max}\right]$$

This piecewise formulation explicitly accommodates both deterministic and uncertainty-aware prediction regimes. In the interval-based case, feasibility is determined by verifying the existence of at least one valid combination of predicted endpoint values satisfying the spatial constraint, thereby improving robustness under conditions of elevated prediction uncertainty.

#### 3.5.3. Overlap Screening

Let $T_{ij}$ denote the set of timestamps shared by trajectory segments $\tau_{i}$ and $\tau_{j}$:

(17) $$T_{ij}=\left\{t|t\in \tau_{i}\cap \tau_{j}\right\}$$

A candidate pair is admitted to the matching pool only if:

(18) $$\left|T_{ij}\right|\leq N_{max}\;\mathrm{and}\;\underset{t\in T_{ij}}{\max}\left|s_{i}\left(t\right)-s_{j}\left(t\right)\right|\leq \varepsilon_{s}$$

where $N_{max}$ specifies the maximum permissible number of temporally overlapping observation points and ϵ_s_ defines the spatial tolerance for overlap assessment. This constraint accommodates limited temporal co-occurrence attributable to sensing noise while precluding the erroneous association of trajectory segments originating from distinct physical vehicles.

#### 3.5.4. One-to-One Matching Constraint Enforcement

To preserve the physical integrity of reconstructed trajectories, each trajectory segment is constrained to participate in at most one matching pair. Upon selecting a valid match for a given segment, the candidate segment is immediately excluded from consideration in all subsequent matching evaluations. This constraint eliminates ambiguous multi-assignment configurations, such as multiple segments connecting to a common endpoint, and ensures that the resulting reconstructed trajectories remain physically plausible and interpretable.

The parameter values governing the proposed framework are as follows. The temporal threshold $T_{max}$ = 2.5 s reflects the maximum admissible duration of trajectory interruption under typical traffic conditions. At the study site on E 2nd Street, the posted speed limit is 35 mph (approximately 15.6 m/s); however, vehicles approaching the signalised intersection typically travel at reduced speeds due to deceleration, queuing, and signal control, with observed upstream operating speeds averaging approximately half the posted limit, i.e., approximately 7.8 m/s (17.5 mph). At this representative operating speed, a vehicle would traverse approximately 19.5 m during a 2.5 s occlusion interval, which is directly consistent with the spatial threshold $S_{max}$ = 20 m and represents a physically plausible upper bound on the gap duration before a fragmented trajectory can no longer be reliably attributed to the same vehicle. Furthermore, when negative velocities are detected within the five-point sliding window during endpoint prediction, indicating local deceleration or queuing, the temporal threshold is adaptively extended by 1 s to $T_{max}$ = 3.5 s. This extension is physically grounded in the observed queue discharge conditions: at the representative upstream operating speed of 7.8 m/s, one additional second corresponds to approximately 7.8 m of additional spatial displacement, sufficient to accommodate the inter-vehicle gap under queued conditions near the signalized intersection. The boundary tolerance δ = 0.2 m is calibrated to discriminate between genuine occlusion-induced truncations and normal detection zone boundary events, representing less than 0.4% of the 50 m detection zone length and therefore negligible relative to typical vehicle displacements. The overlap constraint parameters $N_{max}$ = 3 and $\epsilon_{s}$ = 5 m permit limited temporal co-occurrence attributable to residual sensing noise; at 10 Hz sampling, three overlapping frames correspond to a 0.3 s window. Under the most demanding scenario, in which upstream vehicles are queued and nearly stationary while an opposing lane vehicle travels at the posted speed limit of 35 mph (15.6 m/s), the occluding vehicle traverses approximately 4.7 m within this 0.3 s window, remaining within the 5 m spatial tolerance. This confirms that the overlap constraint is physically grounded in the worst-case relative motion scenario observed at the study site, where the maximum spatial displacement within the overlap window does not exceed $\epsilon_{s}$ regardless of the traffic state of the queued vehicle. All parameter values were grounded in the observed spatiotemporal characteristics of the study site. The temporal threshold $T_{max}$ and spatial threshold $S_{max}$ were further validated through the precision-recall trade-off analysis presented in Section 4.6, which confirms that the selected values correspond to the optimal F1-score on the validation dataset.

Once a valid trajectory pair ($\tau_{i}$, $\tau_{j}$) has been confirmed through the four screening stages, the two segments are stitched into a single continuous trajectory. The terminal observations of $\tau_{i}$ and the initial observations of $\tau_{j}$ are concatenated in chronological order, and the gap interval between the predicted endpoint of $\tau_{i}$ and the observed head of $\tau_{j}$ is filled via linear interpolation in the time–space domain. The interpolated points are assigned timestamps uniformly distributed within the gap interval, with spatial positions estimated from the mean velocity computed over the prediction window. The stitched trajectory inherits the identifier of $\tau_{i}$, and $\tau_{j}$ is removed from the active trajectory set. The resulting merged segment is treated as a single vehicle observation for all downstream analyses, including vehicle counting, speed estimation, and conflict detection. It should be emphasized that the linearly interpolated gap segment serves solely as a visual representation of the association decision and does not claim to reproduce the true vehicle trajectory during the occlusion interval; the vehicle is by definition unobservable during this period, and geometric fidelity of the interpolated segment is therefore outside the scope of the proposed framework, which is explicitly a trajectory association and count correction method.

## 4. Experimental Validation and Performance Evaluation

### 4.1. LiDAR Trajectory Dataset Generation

**Figure 6:**
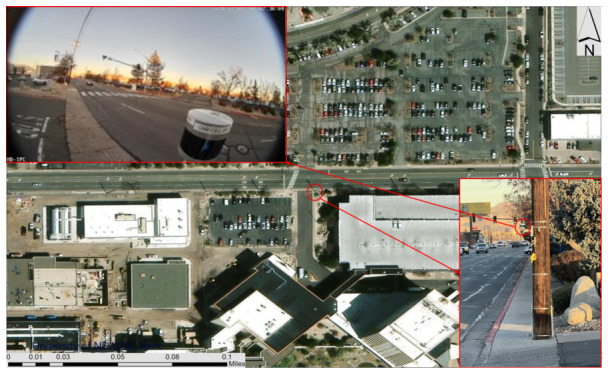
Study area and LiDAR sensor deployment location at E 2nd Street, Reno, Nevada.

The LiDAR sensor was installed at the southeast corner of the signalized intersection between E 2nd Street and an adjacent parking lot driveway access point in Reno, Nevada, USA, at a location approximately 440 ft (134 m) west of the E 2nd Street and Manuel Street intersection, as shown in Figure 6. E 2nd Street functions as the primary east–west arterial with an asymmetric lane configuration: one westbound through lane; two eastbound approach lanes; a shared two-way left turn lane (TWLTL) in the median; and a two-way driveway access lane on the minor road connecting to adjacent parking lots and commercial land uses to the north and south. LiDAR data were collected across multiple representative traffic periods, including the weekday PM peak (11 June 2025, 16:00–19:00), weekday AM peak (12 June 2025, 08:00–09:00), weekend AM peak (14 June 2025, 09:00–10:00), and weekend PM peak (14 June 2025, 17:00–18:00). For performance evaluation, a 30 min sub-period was selected from each collection session as the primary analysis window: 16:00–16:30 for the weekday PM peak, 08:00–08:30 for the weekday AM peak, 09:00–09:30 for the weekend AM peak, and 17:00–17:30 for the weekend PM peak. The point cloud sampling interval was 0.1 s, and the effective vehicle detection range was approximately 150 m.

Raw LiDAR point clouds were processed using a baseline vehicle detection and tracking framework to identify individual vehicles and reconstruct their movement trajectories. Each detected vehicle was continuously tracked across consecutive LiDAR frames to produce a complete trajectory representation [19]. The resulting dataset was structured at the trajectory-segment level, with key spatiotemporal attributes recorded for each segment, including vehicle presence duration, traveled distance within the detection zone, and zone entry and exit timestamps. Detected road users were classified into three categories, namely pedestrians, vehicles, and micromobility users, based on geometric features estimated from the point cloud data. All trajectory data were exported and stored in comma-separated value (CSV) format for subsequent processing and analysis.

### 4.2. Dataset and Evaluation Protocol

**Figure 7:**
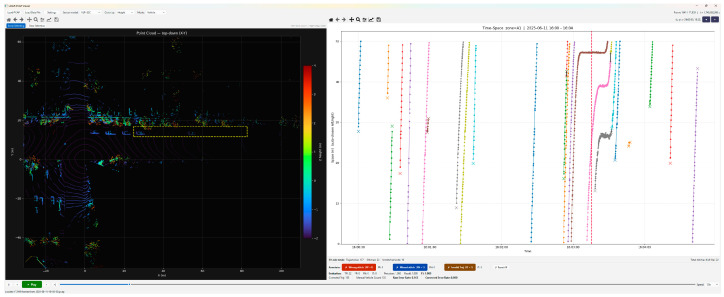
Custom graphical interface for trajectory stitching validation and ground truth annotation.

A custom desktop application was developed to facilitate interactive ground truth annotation and visual verification of trajectory reconstruction results (Figure 7). The application integrates two synchronized panels within a unified graphical interface: a top-down LiDAR point cloud viewer on the left and a time–space diagram on the right. A left-click on any position in the time–space diagram automatically navigates the PCAP file to the closest matching LiDAR frame, enabling frame-level inspection of individual occlusion events and direct visual confirmation of each stitching decision.

The point cloud panel renders decoded LiDAR frames as top-down scatter plots with Z-height color mapping, supporting right-drag rotation and Ctrl + scroll zoom for geometric inspection from multiple viewpoints. The time–space panel organizes trajectory observations into fixed-duration time slots, automatically identifies the dominant travel direction by comparing cumulative lateral displacements, and renders each trajectory as a colored polyline with predicted endpoints marked as either a single point or an interval-based ray depending on the velocity consistency within the prediction window. Matched trajectory pairs are visualized as linearly interpolated segments connecting the predicted tail and head endpoints, where the interpolation serves as a visual representation of the association decision rather than a claim of geometric fidelity during the occlusion interval, allowing analysts to immediately distinguish correctly stitched chains from isolated fragments. To support spatial verification of detection zone placement, the point cloud panel provides a zone selection function accessible via the Select Zone button in the toolbar. When activated, the analyst draws a rectangular region directly over the point cloud by left-dragging the mouse; the selected region is rendered as a yellow dashed rectangle overlay and propagated to the time–space panel as the active detection zone. This functionality enables the analyst to visually confirm that the detection zone boundaries align with the intended lane geometry in the LiDAR coordinate frame, and to adjust the zone interactively before committing to a full trajectory extraction run. Once the zone is confirmed, the Clear Selection button removes the overlay and restores standard pan and zoom navigation.

Ground truth labels were established through manual annotation directly within the application. For each inspected slot, analysts reviewed every linearly interpolated association link against the corresponding LiDAR frame and annotated false positive stitches, missed stitches, and invalid trajectories using dedicated buttons. This frame-synchronized workflow ensured that annotation decisions were grounded in direct observation of the underlying point cloud geometry rather than trajectory data alone, producing reliable ground truth for all benchmark comparisons reported in Section 4.3.

### 4.3. Performance Evaluation

**Figure 8:**
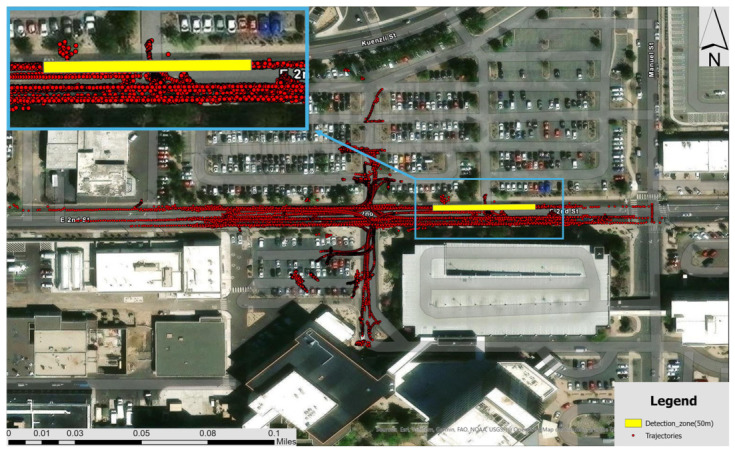
Vehicle trajectories and 50 m-long lane detection zone used for occlusion analysis.

To assess the effectiveness, robustness, and generalizability of the proposed trajectory discontinuity correction method, a 50 m-long lane detection zone was adopted as the primary evaluation configuration to encompass more complex occlusion conditions (Figure 8). For the purposes of this evaluation, precision is defined as the proportion of correctly identified trajectory matches among all proposed matches, reflecting the method’s ability to avoid false associations; recall quantifies the proportion of true discontinuities that are successfully detected and recovered; and the F1-score is their harmonic mean, providing a balanced measure of overall reconstruction performance.

**Figure 9:**
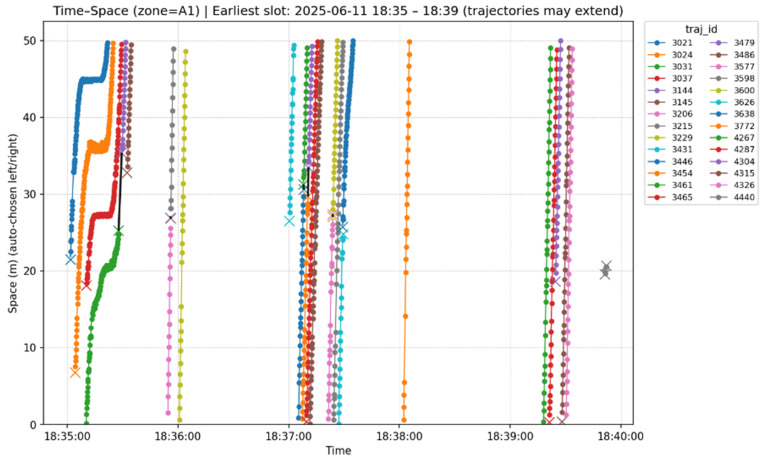
Time–space diagram following trajectory discontinuity correction for the 50 m-long lane detection zone. The symbol “×” denotes valid occlusion-induced endpoint predictions; solid black connecting lines indicate successfully corrected trajectory pairs.

Figure 9 presents the corrected time–space diagram for the 50 m detection zone, where the symbol ‘×’ marks valid occlusion-induced endpoint predictions and the solid black lines indicate successfully stitched trajectory pairs, visually confirming the effectiveness of the correction procedure. For the Weekday PM Peak 30 min sub-period (16:00–16:30), manual verification identified 135 individual vehicles against 160 raw trajectories, representing an uncorrected count error of 18.5%. Following correction, 22 fragmented trajectories were successfully stitched, yielding 138 corrected trajectories and reducing the count error to 2.2%.

Table 1 summarizes the performance of the proposed trajectory correction method under four representative 30 min peak-period datasets, covering weekday and weekend AM and PM peak conditions. Trajectory reconstruction performance is evaluated using precision, recall, and F1-score, while vehicle counting performance is assessed through the raw and corrected count error rates. The results demonstrate that the proposed method consistently achieves high reconstruction accuracy across different traffic conditions and substantially reduces vehicle count errors caused by occlusion-induced trajectory fragmentation. Overall, the corrected count error is substantially lower than the raw count error for all periods, confirming the effectiveness and robustness of the proposed correction framework.

It should also be noted that traffic volumes during weekend peak periods were noticeably lower than those during weekday peak periods. Consequently, fewer occlusion-induced trajectory discontinuities occurred, resulting in a reduced number of trajectory stitching operations and a smaller trajectory dataset overall. The lower traffic density decreased the frequency of trajectory fragmentation, leading to substantially lower raw count error rates (6.2–6.8%) compared with those observed during weekday peak periods (18.5–26.4%).

### 4.4. Comparison of Post-Processing Procedures

A benchmark comparison was conducted using a dataset containing 139 fragmented trajectories and 5999 frames collected during the 08:00 to 08:30 period on June 12 within the 50 m-long lane detection zone. The proposed method was compared against three representative trajectory stitching approaches: Interacting Multiple Model filtering (IMM), Graph Neural Network with Relation Matching (GNN-RM), and Hidden Markov Model combined with the Viterbi algorithm (HMM + Viterbi). Evaluation results are summarized in Table 2.

IMM is a probabilistic state estimation framework that runs multiple Kalman filters in parallel, each representing a distinct vehicle motion mode such as constant velocity and constant acceleration and combines their outputs through mode probability weighting to predict vehicle positions during occlusion gaps [20]. GNN-RM is a deep learning-based trajectory completion method that constructs a graph representation of trajectory fragments, extracts spatial interaction features between vehicles through graph neural network layers, and scores candidate trajectory associations using a multi-head attention regeneration module [21]. HMM + Viterbi models trajectory fragment association as a probabilistic sequence problem, where hidden states represent candidate matching pairs and emission probabilities encode spatiotemporal compatibility; the Viterbi algorithm then identifies the globally optimal matching sequence through dynamic programming [22]. Unlike the proposed method, which applies deterministic spatiotemporal constraints without requiring training data or motion model calibration, these three approaches represent probabilistic, learning-based, and optimization-based paradigms, respectively, providing a comprehensive benchmark across different methodological categories.

The proposed method achieves the best overall performance with an F1-score of 0.980. It attains perfect precision, indicating that all stitched trajectory pairs are correctly associated, while only one valid trajectory pair remains unrecovered. The runtime of 1.54 s demonstrates that the method maintains high reconstruction accuracy while remaining computationally efficient. This performance is primarily attributed to the explicit incorporation of spatiotemporal continuity constraints and physically interpretable matching criteria tailored to the roadside LiDAR sensing environment.

Among the benchmark methods, IMM provides the strongest competing performance, achieving balanced precision and recall values of 0.880. Its runtime of 1.63 s is comparable to that of the proposed method, indicating favorable computational efficiency. The relatively stable performance suggests that IMM generalizes effectively under moderate trajectory fragmentation conditions, making it a viable alternative when training data are unavailable.

GNN-RM achieves lower reconstruction performance despite significantly higher computational cost, with an F1-score of 0.792 and a runtime of 4.75 s. Although the method leverages graph-based trajectory representations to capture spatial interaction features, its performance is constrained by the relatively small training dataset used in this study. Since the graph neural network is trained using pseudo-ground-truth trajectory associations, the limited number of fragmented trajectories restricts its ability to learn robust trajectory representations. Substantially larger training datasets would likely be required to realize the full potential of this approach.

The HMM + Viterbi approach achieves the highest computational efficiency, requiring only 0.03 s for processing. However, its recall decreases substantially to 0.600, indicating that a large proportion of valid trajectory discontinuities remain unrecovered. This behavior suggests that the Viterbi-based optimization adopts conservative stitching decisions, prioritizing high-confidence isolated associations over uncertain candidate pairs. Consequently, while HMM + Viterbi is well suited to real-time applications with strict computational constraints, its reconstruction completeness remains limited under complex occlusion conditions.

**Figure 10:**
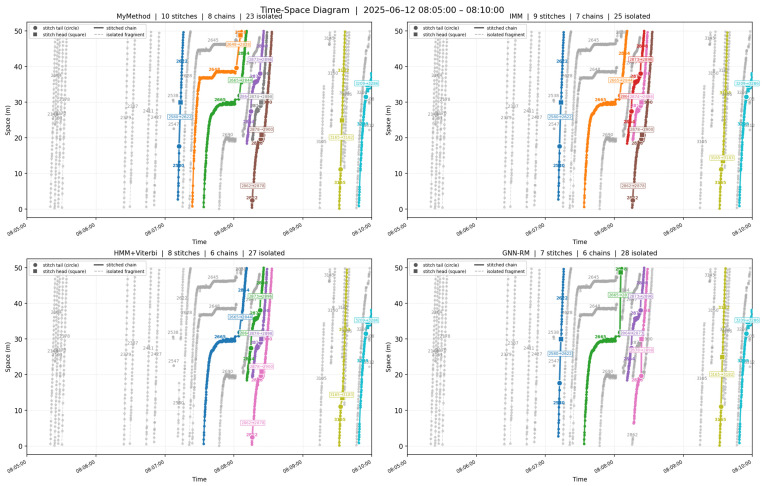
Time–space diagrams of trajectory reconstruction results for the 08:05–08:10 interval. Solid lines denote stitched trajectory chains; dashed lines denote isolated fragments. Circle and square markers indicate stitch tail and head endpoints, respectively.

Figure 10 presents the time–space diagrams produced by each method for the 08:05 to 08:10 interval, providing a qualitative illustration of the reconstruction differences. The proposed method recovers the greatest number of stitched pairs (10 stitches, 8 chains, 23 isolated fragments), successfully linking fragmented trajectories across the full spatial extent of the detection zone including both short-gap and long-gap occlusion events. IMM produces comparable results, with most major trajectory chains correctly reconstructed; however, one additional fragment remains unrecovered relative to the proposed method, reflecting its occasional inability to resolve ambiguous candidate pairs under velocity uncertainty. HMM + Viterbi recovers fewer stitches, consistent with its conservative association strategy; several trajectory pairs that are visually continuous in the time–space diagram are left unstitched, confirming the recall limitation observed in the quantitative results. GNN-RM produces the fewest stitches despite forming a similar number of chains to IMM, indicating that while its graph-based representations capture some structural associations correctly, a number of valid pairs are missed or incorrectly scored due to limited training data. Overall, the time–space diagrams confirm that the proposed method produces the most complete and spatially coherent trajectory reconstruction among all evaluated approaches.

From a computational perspective, the theoretical complexity of the proposed method is $O\left(N\right)$ in the number of candidate fragment pairs, reflecting its sequential one-pass matching strategy in which each trajectory is evaluated against eligible candidates exactly once. By contrast, IMM has complexity $O\left(N\cdot K\cdot T\right)$, where K is the number of motion models and *T* is the gap duration in timesteps; HMM + Viterbi has complexity *O*(*N*^2^ · *T*) due to the Viterbi dynamic programming over all candidate pair combinations; and GNN-RM has complexity $O\left(N\cdot d\cdot L\right)$ where d is the node feature dimension and L is the number of graph neural network layers. The empirical runtimes reported in Table 2 are consistent with these theoretical estimates: the proposed method (1.54 s) and IMM (1.63 s) exhibit comparable and favorable efficiency, while GNN-RM incurs substantially higher cost (4.75 s) due to graph construction and neural network inference. HMM + Viterbi achieves the fastest runtime (0.03 s) owing to its compact state space, though this comes at the cost of reconstruction completeness as discussed above. Overall, the proposed method achieves the best balance between reconstruction accuracy and computational efficiency among all evaluated approaches, confirming its practical suitability for deployment in real-time traffic monitoring pipelines.

Overall, the comparison demonstrates that the proposed method provides the best balance among reconstruction accuracy, robustness, and computational efficiency for roadside LiDAR trajectory discontinuity correction. IMM offers competitive performance without requiring training data; HMM + Viterbi provides extremely fast processing speed suitable for real-time deployment; and GNN-RM represents a promising direction contingent on the availability of larger annotated datasets. The proposed method achieves superior trajectory reconstruction quality across all evaluated metrics, confirming its suitability for occlusion-affected traffic monitoring environments.

### 4.5. Comparison Across Different Scales

**Figure 11:**
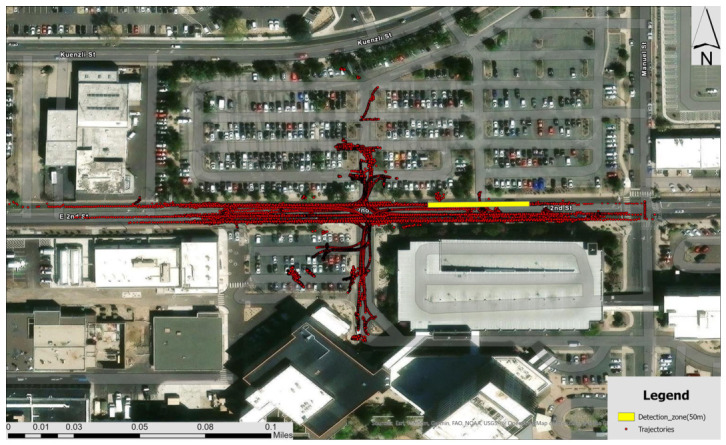
Vehicle trajectories and 150 m-long lane detection zone used for occlusion analysis.

To systematically examine the influence of detection zone spatial scale on time–space representation characteristics and correction performance, lane detection zones of 10 m, 50 m, and 150 m in length were evaluated comparatively. The 150 m zone was positioned with its center at the main signalized intersection, simultaneously encompassing both the intersection and an adjacent driveway access point (Figure 11), thereby introducing a diverse range of complex traffic behaviors, including vehicle deceleration and stopping, acceleration, turning maneuvers, and potential lane changes.

As summarized in Table 3, the raw vehicle count error rate increases markedly with lane detection zone length, rising from 2.5% for the 10 m zone to 35.8% for the 150 m zone. This trend reflects the progressive amplification of occlusion-induced trajectory fragmentation as the spatial extent of the monitored region increases. Following application of the proposed correction method, vehicle counts exhibit consistently improved agreement with manual ground truth observations across all spatial scales. The most substantial correction gain is achieved for the 50 m-long lane detection zone, where the error rate is reduced from 18.5% to 2.2%, indicating that most discontinuities at this scale are attributable to brief occlusion events that are reliably identified and corrected within the time–space representation.

For the 10 m-long lane detection zone, improvement is marginal, as the baseline trajectory extraction algorithm already achieves high counting accuracy at this scale. For the 150 m-long lane detection zone, the correction method continues to yield positive improvements; however, performance gains are comparatively attenuated. This reduction in effectiveness can be attributed to two primary factors. First, the extended detection zone spans the signalized intersection, where vehicle trajectories exhibit substantially more complex behaviors than those observed within midblock segments. Vehicles traveling along the monitored lane frequently perform lane changes and turning maneuvers within the intersection area, while vehicles crossing the intersection from the south may temporarily enter the detection zone before leaving it shortly afterward. These behaviors generate a large number of trajectory endpoints in the vicinity of the intersection, increasing the likelihood of incorrect trajectory associations during the stitching process.

**Figure 12:**
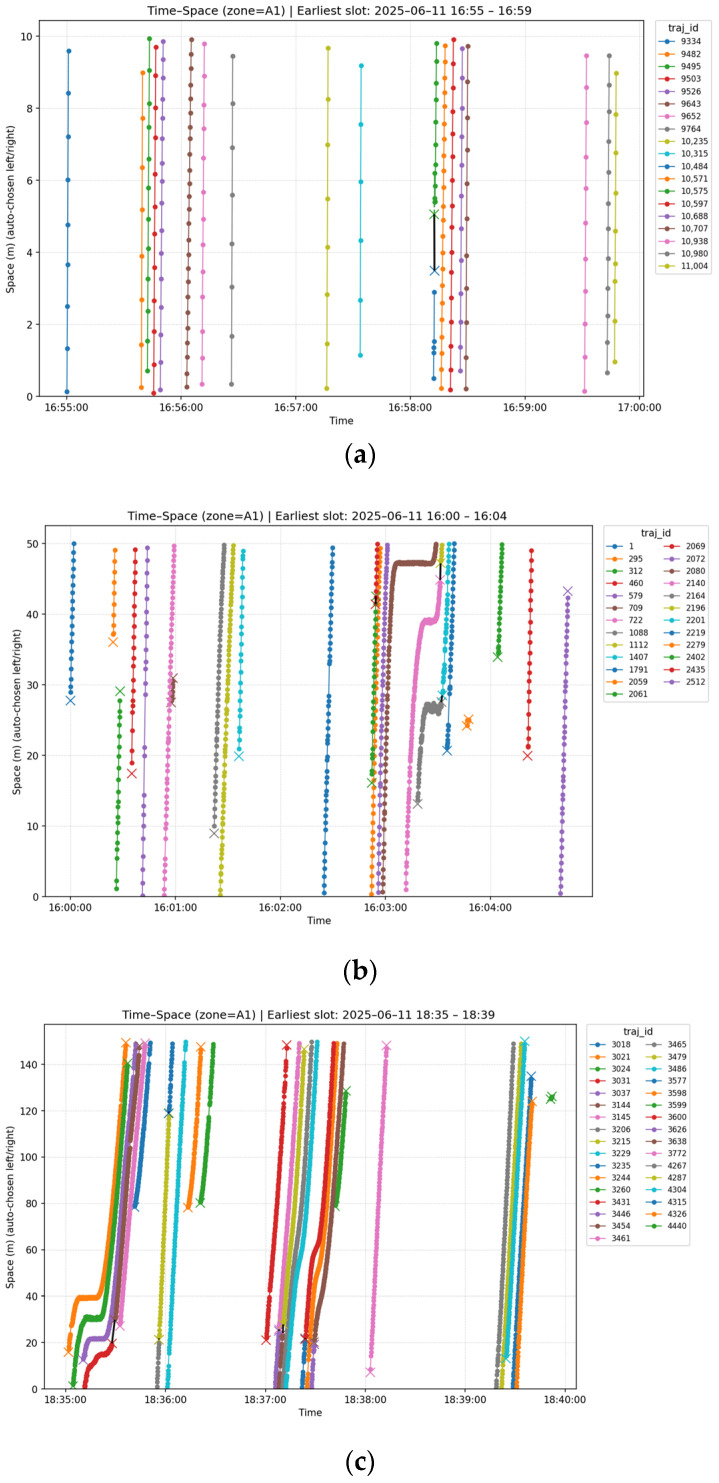
Corrected time–space diagrams for lane detection zones of (**a**) 10 m, (**b**) 50 m, and (**c**) 150 m in length.

Second, the highly dynamic traffic conditions near the intersection introduce additional challenges for the baseline trajectory generation algorithm. Frequent vehicle interactions, turning movements, and trajectory overlaps increase the occurrence of invalid or erroneous trajectories that do not result from occlusion-induced fragmentation. Since the proposed method is specifically designed to reconnect valid trajectory segments through endpoint matching in the time–space domain, errors originating from invalid trajectory generation cannot be effectively corrected through the stitching process. Consequently, a portion of the residual count error in the 150 m detection zone remains attributable to trajectory extraction limitations rather than trajectory discontinuities themselves (see Figure 12).

Among the evaluated configurations, the 50 m-long lane detection zone provides the most favorable combination of spatiotemporal observability and correction robustness, offering clearly resolvable trajectory patterns while remaining largely unaffected by the behavioral complexity that characterizes longer lane detection zones.

### 4.6. Precision–Recall Trade-Off Analysis

**Figure 13:**
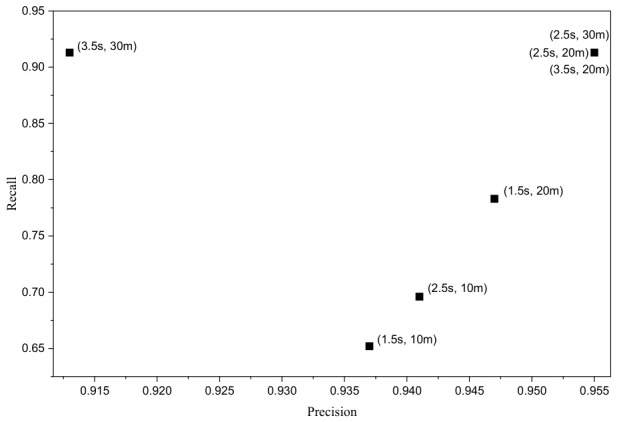
Precision–recall trade-off curve under varying temporal and spatial threshold configurations.

A systematic precision-recall trade-off analysis was performed by varying $T_{max}$ and $S_{max}$ across a range of values while holding all other parameters fixed, with results presented in Figure 13. The analysis confirms the expected inverse relationship between precision and recall: tighter constraints yield higher precision at the expense of reduced recall, as fewer but more certain trajectory associations are proposed; conversely, relaxed constraints recover a greater proportion of true discontinuities while admitting a higher rate of erroneous associations.

Two key findings from the analysis directly justify the selected parameter values. First, moderately relaxing either $T_{max}$ or $S_{max}$ in isolation produces only marginal changes in both precision and recall, demonstrating that the matching framework is robust to individual parameter perturbations within a physically meaningful range. Second, coordinated relaxation of both thresholds simultaneously produces a more pronounced reduction in precision without corresponding recall gains, indicating that the selected values of $T_{max}$ = 2.5 s and $S_{max}\;$= 20 m represent the point beyond which further threshold expansion does not confer additional reconstruction performance. The selected configuration therefore corresponds to the optimal F1-score on the validation dataset, confirming that the physically derived values established in Section 3.5.4 are consistent with the empirically optimal configuration identified through the trade-off analysis.

## 5. Conclusions

This study presents an occlusion-aware trajectory discontinuity correction framework for roadside LiDAR-based traffic monitoring, grounded in time–space analysis. Trajectory extraction from roadside LiDAR deployments is frequently compromised by occlusion and sensor noise, yielding fragmented trajectory records and introducing systematic bias into downstream traffic analyses including vehicle counts, speed estimation, and conflict detection. The proposed framework addresses these limitations through three key components: spatiotemporal trajectory representation, which transforms raw LiDAR-derived segments into lane-specific time–space diagrams; occlusion-aware discontinuity identification, which discriminates genuine occlusion-induced truncations from natural detection zone boundary events through endpoint prediction and boundary threshold validation; and constrained trajectory matching and stitching, which enforces spatiotemporal consistency, occlusion eligibility, overlap screening, and one-to-one matching to produce physically plausible reconstructed trajectories.

Validation on real-world LiDAR data collected at a signalized urban intersection in Reno, Nevada, demonstrates consistent reconstruction performance across varying traffic demand conditions. Across four evaluated 30 min peak-period datasets covering AM and PM conditions on both weekdays and weekends, the proposed method achieves an average precision of 0.989, average recall of 0.916, and average F1-score of 0.948, with an average corrected vehicle count error of 2.6%. For the Weekday PM Peak period, vehicle count error is reduced from 18.5% to 2.2%, confirming the method’s effectiveness in achieving its primary objective of correcting occlusion-induced fragmentation bias. Benchmark comparison against IMM, GNN-RM, and HMM + Viterbi demonstrates that the proposed method achieves the best overall balance among reconstruction accuracy, robustness, and computational efficiency, with an F1-score of 0.980 and a runtime of 1.54 s. The analysis further establishes that correction performance is sensitive to detection zone spatial scale: a moderate scale of 50 m provides the most favorable balance between spatiotemporal observability and reconstruction robustness, while excessively long zones introduce complex behavioral phenomena including lane changes and extended queue discharge near signalized intersections that attenuate correction effectiveness.

The current framework is subject to several limitations. The validation is conducted at a single urban signalized intersection under fair weather conditions, and empirical confirmation across multiple sites, sensor models, adverse weather conditions, and diverse geometric configurations has not been conducted and represents a primary direction for future work. While LiDAR sensing offers inherent robustness to low-light and moderate weather variations compared with camera-based systems, extreme weather conditions such as heavy rain, dense fog, or snow may degrade upstream point cloud quality and adversely affect the trajectory fragments supplied to the post-processing framework. In particular, under extreme deceleration or turning maneuvers near the signalized intersection, the interval-based endpoint prediction may produce spatial ranges that are too narrow to capture the true endpoint position, potentially causing matching failures; learning-based endpoint prediction represents a promising future direction to better capture non-linear motion patterns under such conditions. Furthermore, the current framework is validated exclusively on motorized vehicle trajectories, as the study site did not provide sufficient non-motorized vehicle volume for systematic evaluation; extension to non-motorized vehicles such as bicycles and motorcycles would require recalibration of the spatiotemporal threshold parameters and improvements to upstream tracking performance for small and sparse point cloud targets. Future work will pursue adaptive parameter learning, integration of macroscopic traffic constraints, and learning-based endpoint prediction to enhance generalizability across diverse operational environments. Extensions to multi-sensor fusion architectures, non-motorized vehicle scenarios, and large-scale urban road network deployments will also be investigated. The proposed method constitutes a practical, computationally efficient, and physically interpretable solution for enhancing trajectory data quality in roadside LiDAR-based traffic monitoring applications.

## Figures and Tables

**Table 1:** Performance evaluation results across four representative 30 min peak-period datasets covering weekday and weekend AM and PM peak conditions.

Traffic Period	Precision	Recall	F1	Raw Error Rate (%)	Corrected Error Rate (%)
Weekday AM Peak	1.000	0.960	0.980	26.4	5.7
Weekday PM Peak	0.955	0.955	0.955	18.5	2.2
Weekend AM Peak	1.000	1.000	1.000	6.8	0
Weekend PM Peak	1.000	0.750	0.857	6.2	2.5
Average	0.989	0.916	0.948	14.5	2.6

**Table 2:** Performance comparison of post-processing procedures. Note: the benchmark comparison in Table 2 was conducted on the 08:00–08:30 sub-period of the Weekday AM Peak dataset.

Method	Precision	Recall	F1	Runtime
Proposed Method	1.000	0.960	0.980	1.54 s
IMM	0.880	0.880	0.880	1.63 s
GNN-RM	0.826	0.760	0.792	4.75 s
HMM + Viterbi	0.833	0.600	0.698	0.03 s

**Table 3:** Vehicle count comparison before and after trajectory correction across lane detection zones of different lengths.

Metric	10 m	50 m	150 m
Time interval	16:00–16:30	16:00–16:30	16:00–16:30
Raw trajectory count	122	160	205
Corrected trajectory count	121	138	159
Manual vehicle count	119	135	151
Raw error rate	2.5%	18.5%	35.8%
Corrected error rate	1.7%	2.2%	5.3%

## Data Availability

The data presented in this study are available on request from the corresponding author.

## Abbreviations

The following abbreviations are used in this manuscript:

LiDAR	Light Detection and Ranging
TWLTL	Two-Way Left Turn Lane
CSV	Comma-Separated Value
IMM	Interacting Multiple Model
GNN-RM	Graph Neural Network with Relation Matching
HMM	Hidden Markov Model
GPS	Global Positioning System
GNN	Global Nearest Neighbor
MHT	Multiple Hypothesis Tracking
JPDA	Joint Probabilistic Data Association
